# Assessment of Cognitive Function in European Adults Aged 50+in Relation to Their Handgrip Strength and Physical Inactivity: The SHARE Study During 2019-2020

**DOI:** 10.34172/jrhs.2024.146

**Published:** 2024-06-01

**Authors:** Nikos Rikos, Manolis Linardakis, Emmanouil Smpokos, Eleni Spiridaki, Emmanouil K Symvoulakis, Ioanna Tsiligianni, Anastas Philalithis

**Affiliations:** ^1^Department of Nursing, School of Health Sciences, Hellenic Mediterranean University, Heraklion, Greece; ^2^Department of Social Medicine, Faculty of Medicine, University of Crete, Greece

**Keywords:** Cognitive function, Cognitive impairment, Handgrip strength, Physical inactivity, SHARE study

## Abstract

**Background:** Cognitive function is crucial during aging. This study assessed the cognitive function of European adults aged 50 and over in relation to handgrip strength and physical inactivity.

**Study Design:** This was a cross-sectional survey.

**Methods:** Data were collected from 41,395 adults from 27 European countries participating in the Survey of Health, Ageing, and Retirement in Europe (SHARE) during 2019-2020. Cognitive function was assessed based on five tests, and cognitive impairment was defined using 3+tests. Handgrip strength and physical inactivity were also correlated through the analysis of covariance using a complex study design.

**Results:** The majority of participants were female (56.6%), with a mean age of 70.9 years, and 22.6% presented multimorbidity. Furthermore, 51.1% had a normal cognitive function, while 13.3% had cognitive impairment (The estimated population was 21,944,722). Moreover, cognitive impairment was more prevalent in females than in males (14.4% vs. 12.0%, *P*<0.001) in patients with no years of education (*P*<0.001) and origin from southern European countries (*P*<0.001). Additionally, participants with cognitive impairment had lower mean handgrip strength compared to those with cognitive impairment in 1-2 criteria or with normal cognitive function (29.3 vs. 33.4 and 35.1 kg, respectively, *P*<0.001). Physically inactive participants had higher odds ratio (OR) of cognitive impairment than those engaging in moderate/vigorous physical activity, both in 1-2 tests (OR:1.73, 95% confidence interval (CI): 1.32-2.26) and in 3+tests (OR: 3.36, 95% CI: 2.57-4.40).

**Conclusion:** Cognitive impairment presented low prevalence and was associated with low levels of handgrip strength and physical inactivity. These specific factors may play a special role in early detection, diagnosis, and treatment or may slow down the progression of cognitive impairment.

## Background

 The ancient Greek tragedian Euripides (480-406 BC) said ‘*Δεινόν το γῆρ ας, οὐ γάρ ἔρχετ αι μόνον*’ (‘*old age is terrible for it does not come alone*’). Aging often leads to a decrease in cognitive and physical function. This can result in weakness, limitations, or even loss of independence.^1,2^ Regarding cognitive function, mild cognitive impairment (MCI) has recently been defined as memory loss that occurs at a higher level than that normally associated with aging but does not interfere with daily activities. It is essentially a transitional stage between normal cognitive function and senility.^3^

 Good cognitive function in middle-aged people is an important factor related to health and quality of life in later old age while maintaining independence in daily life.^4^ However, MCI is the early stage of cognitive impairment between the expected cognitive decline due to aging and dementia.^5^ Alzheimer’s disease, for example, was recognized in the United States in 5.8 million people over 65 years of age, while deaths increased by 146.2% between 2000-2018.^6^ Globally, cognitive impairment ranges from 5.1% to 41.0%, with a median frequency of 19.0%.^7^ Alternatively, the prevalence of cognitive impairment is reported to approach 23 and 9 per 1000 person-years for mild and severe impairment, respectively.^8^

 Nevertheless, it seems important to identify the predictive risk factors for impending cognitive decline. It appears that age, low education level, low income, reduced physical activity or physical inactivity, and chronic diseases such as diabetes, depression, and hypertension contribute to reduced cognitive function.^9-11^ Furthermore, as an indicator of physical/muscle weakness, reduced handgrip strength is reported to be associated with poorer memory, slower elaborating speed, and reduced working memory.^12,13^ Handgrip strength is generally an easily and reliably measurable indicator of general health in middle and old age that has later been linked to a higher risk of dementia.^14^

 Thus, the current study aimed to assess the cognitive function of adults aged over 50 in European countries in relation to handgrip strength and physical inactivity.

## Methods

###  Study population and sample

 In the present study, data from Wave 8 of the Survey of Health, Ageing and Retirement in Europe (SHARE) (https://share-eric.eu/) were collected during 2019-2020 from 27 European countries (Austria, Belgium, Bulgaria, Croatia, Cyprus, Czech Republic, Denmark, Estonia, Finland, France, Germany, Greece, Hungary, Italy, Latvia, Lithuania, Luxembourg, Malta, Netherlands, Poland, Romania, Slovakia, Slovenia, Spain, Sweden, Switzerland, and Israel). The survey included 46 733 people aged 32-104 or 46,547 aged 50 and over. Detailed information about composite sample selection, weights, ethical standards, data collection, interviews, and the like are described in previous reports.^15,16^ The final analysis sample size of the present study amounted to 41,395 people aged 50 + , representing a target population of 165 044 028 people based on selection weights. This was due to unavailable data and the exclusion of 1162 people (2.5%) diagnosed with acquired impairment of mental abilities such as Alzheimer’s disease, dementia, and senility

 The SHARE study was conducted according to the guidelines of the Declaration of Helsinki and approved by the Ethics Council of the Max Planck Society. All participants gave their informed consent to be included in the study.^15^

###  Cognitive function, handgrip strength, and physical inactivity

 Cognitive function was evaluated using five different tests: verbal fluency, immediate recall, delayed recall, and numeracy and orientation.^17,18^ The verbal fluency score was derived from an animal-naming fluency test in which participants were asked to name as many animals as they could think of in one minute, and its score was the sum of the accepted animals. The immediate recall of 10 words and delayed recall were derived from an adapted 10-word delayed recall test. Their score (range 0-10) was the number of words recalled after the researcher read a list of 10 words from their computer screen. At the end of the cognitive testing session, participants were again asked to recall any of these words while the researcher recorded the total number of recalled words (0-10). The numeracy test deals with mathematical performance and is measured via nine items in the SHARE study, 5 items measuring subtraction calculation skills and 4 items measuring percentage calculation skills.^18^ The resulting score is based on the number of correct responses, with a higher score indicating better numeracy or mathematical performance (range from 0: bad to 5: good). Four items in the SHARE study assess participants’ orientation to date, month, year, and day of week test, as well as participants’ ability to correctly answer the questions concerning the current day of the month, the month, the year, and the day of the week. The extracted scores range from 0 (bad orientation) to 4 (good), with a higher score indicating a better orientation.^18^

 Since the scores of the five criteria have different degrees and ranges, they can be separated according to predefined value limits (cut-offs) to define low levels of cognitive function or the presence of cognitive impairment. These values were as follows: fewer than 15 animal names in verbal fluency, fewer than 5 words in immediate recall, fewer than 4 words in delayed recall, and a score of 0 and/or 1 for numeracy and orientation.^17,18^ The participants with values within each of the above limits were assigned a score of 1, while the rest were assigned a score of 0. Then, the scores for the five criteria were added for each participant, and a total score of cognitive function was determined as a composite score, ranging from 0 to 5. A score of 0 (zero) defines the absence of cognitive impairment in any of the five tests (normal cognitive function), scores of 1-2 indicate cognitive impairment in 1-2 tests and higher, and scores of 3 or higher indicate the presence of cognitive impairment.^17,19^

 For the handgrip strength test, data were obtained for the dominant hand using double measurement of strength in kilograms (kg) for each hand. The maximum grip strength was also assessed, which is also the final parameter of handgrip strength.^18,20^ Finally, for physical inactivity, combined data from the two relevant questions in the Behavioral Risk section were used, involving information on participation in vigorous and/or moderate-intensity activities.^21^ These questions included:“We would like to know about the type and amount of physical activity you do in your daily life. How often do you engage in vigorous physical activity such as sports, heavy housework, or a job that involves physical labor?” and “How often do you engage in activities that require a moderate level of energy such as gardening, cleaning the car, or going on a walk?”. The responses were categorized as follows: 1) More than once a week, 2) Once a week, 3) One to three times a month, and 4) Hardly ever, or never. For the definition of physical inactivity, responses falling into the fourth category (hardly ever or never) were considered and combined.

###  Additional data

 Demographic and social characteristics, as well as information about participants’ health level and status, were also taken into account. They included gender, age (either in years or categorized as 50-59, 60-69, 70-79, 80-89, 90 + years), family status (married, living with parents, and so on), years of education, current occupation status, body weight (according to body mass index, with overweight and obesity defined as 25.0 + kg/m^2^), morbidity based on 14 diagnosed chronic diseases or conditions (heart attack, high blood pressure or hypertension, high blood cholesterol, stroke, diabetes or high blood sugar, chronic lung disease, cancer, stomach or duodenal ulcer – peptic ulcer, Parkinson’s disease, cataracts, hip fracture or femoral fracture, rheumatoid arthritis - osteoarthritis/other rheumatism, and chronic kidney disease). Participants were also classified based on European region (Northern, Central, and Southern).^19,22^

###  Statistical analysis

 Data were analyzed using SPSS software, version 25.0 (IBM Corp). Weights were applied according to the complex multistage stratification sampling design of the study. Frequency distributions of characteristics of 41 395 participants were estimated. In the assessment of the five cognitive function tests, mean levels of names/words and the prevalence of cognitive impairment were estimated according to predefined cut-offs and through complex sample analysis. The prevalence of cognitive impairment, based on three to five tests, was associated with participants’ characteristics using the χ^2^ method. Handgrip strength and physical inactivity were also compared between categories of characteristics using Student’s t-test, analysis of variance, and χ^2^ test. Handgrip strength was assessed according to the three cognitive function levels (normal, impairment in 1-2, or impairment in 3 + tests) using analysis of covariance (polynomial trend). Covariates included gender, age, education, family status, occupation, high body weight, morbidity, and European country regions. Using the same covariates and based on logistic regression analysis, cognitive function levels (impairment in 1-2 or 3 + tests in relation to normal) were also assessed in relation to physical inactivity.

## Results

 Of the total of 41,395 study participants, 24.0% were from the Northern countries of the European continent, 52.1% from the central countries, and 23.9% from the Southern countries (Table 1). Regarding special characteristics, 56.6% were women, with an average age of 70.9 years (± 9.1), while 19.0% were 80-104 years old. Average years of education were 11.9 years (± 4.5), 87.5% were married or in cohabitation, 21.5% had an occupation, 65.4% were overweight/obese, and 22.6% had multimorbidity or 3 + diseases.

**Table 1:** Characteristics of 41 395 Europeans aged 50 + participating in wave 8 of the SHARE Survey (2019-2020)

**Variables**	**Number**	**Percent**
European regions		
Northern	9955	24.0
Central	21,554	52.1
Southern	9886	23.9
Gender		
Male	17,947	43.4
Female	23,448	56.6
Age (year)		
50-59	4387	10.6
60-69	15,039	36.3
70-79	14,136	34.1
80-89	6781	16.5
90-104	1052	2.5
Education (year)		
0	1241	3.0
1-7	5621	13.6
8-12	22,291	54.0
13 +	12,121	29.4
Family status		
Unmarried, divorced, widowed	5165	12.5
Married, living with partner	36,230	87.5
Occupation		
Employed	8907	21.5
Unemployed, retired, household	32,464	78.5
Body weight		
Normal	13,980	34.6
Overweight, obese	26,367	65.4
Morbidity, conditions, or diseases		
None	11,183	27.0
1	11,834	28.6
2	9040	21.8
3 +	9338	22.6

*Note.* SHARE: Survey of Health, Ageing, and Retirement in Europe.

**Figure 1:**
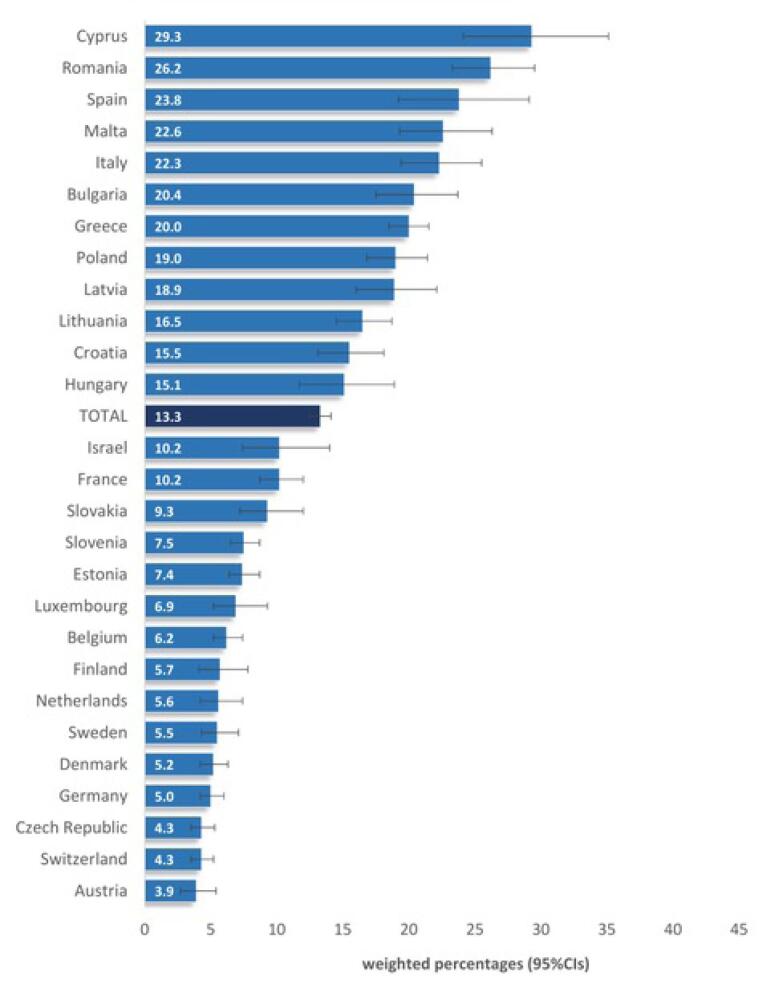
Prevalence of cognitive impairment in 3 + tests of 41 395 Europeans aged 50 + participating in wave 8 of the SHARE survey (2019-2020) in 27 countries. *Note.* SHARE: Survey of Health, Ageing, and Retirement in Europe

 Mean verbal fluency for verbally named animals was 20.7 (± 7.6) within 1 minute, immediate recall of 10 words scored a mean of 5.4 (± 1.7), and delayed recall of 10 words scored a mean of 4.0(± 2.1), as depicted in Table 2. Delayed recall of 10 words was associated with a significantly higher frequency of cognitive impairment (37.9%, 95% confidence interval [CI]: 36.7-39.1), compared to 27.2% (95% CI: 26.1-28.3) for immediate recall of 10 words, 20.6% (95% CI: 19.6-21.5) for verbal fluency, 8.3% (95% CI: 7.6-9.0) for numeracy, and 0.6% (95% CI: 0.5-0.8) for orientation to date, month, year, and day of week. Furthermore, normal cognitive function was observed in 51.1% of participants, cognitive impairment in 20.6% on one test, and 0.4% on five tests, while the prevalence of cognitive impairment (3 + tests) was 13.3% (estimated population N = 21 944 722). Moreover, cognitive impairment was significantly more prevalent in females than in males (results not shown in table) (14.4% *vs.* 12.0%, *P* < 0.001), those aged 90 + years compared to those aged 50-59 (30.4% *vs.* 7.0%, *P* < 0.001), those with no education versus those with 13 + years of education (29.0% *vs.* 6.8%, *P* < 0.001), unmarried/divorced/widowed versus married/living with partner (21.2% *vs.* 12.2%, *P* < 0.001), unemployed/retired/household versus employed (16.2% *vs.* 7.3%, *P* < 0.001), those with 3 + chronic conditions versus those without (19.4% *vs.* 10.5%, *P* < 0.001), and participants from southern versus northern countries (21.9% *vs.* 7.6%, *P* < 0.001). More specifically, among the 27 countries in the study (Figure 1), the highest frequency of cognitive impairment was found in Cyprus (29.3%), followed by Romania (26.2%) and Spain (23.8%), while the lowest was found in Austria (3.9%), Switzerland (4.3%), and the Czech Republic (4.3%).

**Table 2:** Cognitive function assessment tests in 41 395 Europeans aged 50 + and prevalence of cognitive impairment

**Tests**	**Number**	**Estimated population**	**Weighted percentage (95% CI)**
Verbal fluency ^a^			
≥ 15	32 721	131 108 774	79.4 (78.5-80.4)
< 15	8674	33 935 254	20.6 (19.6-21.5)
Immediate recall ^b^			
≥ 5	29 375	120 226 030	72.8 (71.7-73.9)
< 5	12 020	44 817 998	27.2 (26.1-28.3)
Delayed recall ^c^			
≥ 4	24 671	102 448 857	62.1 (60.9-63.3)
< 4	16 724	62 595 171	37.9 (36.7-39.1)
Numeracy score			
2-5	38 196	151 376 83	91.7 (91.0-92.4)
0-1	3199	13 667 445	8.3 (7.6-9.0)
Score of orientation to date			
2-4	41 100	163 998 726	99.4 (99.2-99.5)
0-1	295	1 045 302	0.6 (0.5-0.8)
Clustering of five tests			
None	20 287	84 269 331	51.1 (49.8-52.3)
1	8693	33 987 029	20.6 (19.6-21.6)
2	6716	24 842 946	15.1 (14.1-16.0)
3	4166	15 974 855	9.7 (9.0-10.4)
4	1376	5 385 655	3.2 (3.0-3.6)
5	157	584 212	0.4 (0.3-0.5)
3 +	5699	21 944 722	13.3 (12.5-14.1)

*Note.* CI: Confidence interval. Mean ± standard deviation: ^a^ 20.7 ± 7.6; ^b^ 5.4 ± 1.7; ^c^ 4.0 ± 2.1.

 Handgrip strength had an average value of 33.7 kg (Table 3), with a significantly higher value in younger age groups (*P* < 0.001), those with more years of education (*P* < 0.001), those who were married/living with partner (*P* < 0.001), those without chronic conditions (*P* < 0.001), and those from Northern versus Southern countries (*P* < 0.001). Regarding physical inactivity, 10.8% of participants were inactive, with a higher frequency in older age groups (*P* < 0.001), those with fewer years of education (*P* < 0.001), unmarried/divorced/widowed participants (*P* < 0.001), overweight/obese participants (*P* = 0.041), those with more chronic conditions (*P* < 0.001), and those from Southern countries (*P* < 0.001).

**Table 3:** Handgrip strength levels and physical inactivity in 41 395 Europeans aged 50 + in relation to their characteristics

**Variables**	**Handgrip Strength (kg)**			**Physical Inactivity (%)**		
	**Mean**	**SD**	***P *** **value**	**No**	**Yes**	***P *** **value**
Total	33.7	11.7		89.2	10.8	
Gender			< 0.001			0.299
Male	42.4	9.7		89.6	10.4	
Female	25.9	6.7		88.1	9.0	
Age (year)			< 0.001			< 0.001
50-59	37.7	11.7		95.1	4.9	
60-69	35.2	11.6		90.2	9.8	
70-79	31.7	10.4		87.7	12.3	
80-89	29.6	11.2		83.2	16.8	
90 +	25.4	10.9		73.3	26.7	
Education (year)			< 0.001			< 0.001
0	30.1	11.6		86.2	13.8	
1-7	30.6	11.2		84.4	15.6	
8-12	34.0	11.7		89.0	11.0	
13 +	35.5	11.4		92.3	7.7	
Family status			< 0.001			< 0.001
Unmarried, divorced, widowed	30.4	11.0		85.2	14.8	
Married, with partner	34.2	11.7		89.7	10.3	
Occupation			< 0.001			< 0.001
Employed	37.5	11.8		93.0	7.0	
Unemployed, retired, household	31.9	11.1		87.3	12.7	
Body weight			< 0.001			0.041
Normal	31.8	10.7		90.7	9.3	
Overweight, obese	35.1	11.9		88.8	11.2	
Morbidity, conditions, or diseases			< 0.001			< 0.001
0	35.9	11.3		91.5	8.5	
1	34.7	11.5		91.4	8.6	
2	32.2	11.7		89.3	10.7	
3 +	30.3	11.5		81.8	18.2	
European regions			< 0.001			< 0.001
Northern	35.7	12.1		94.3	5.7	
Central	34.6	11.8		91.9	8.1	
Southern	31.6	10.9		82.5	17.5	

**Figure 2:**
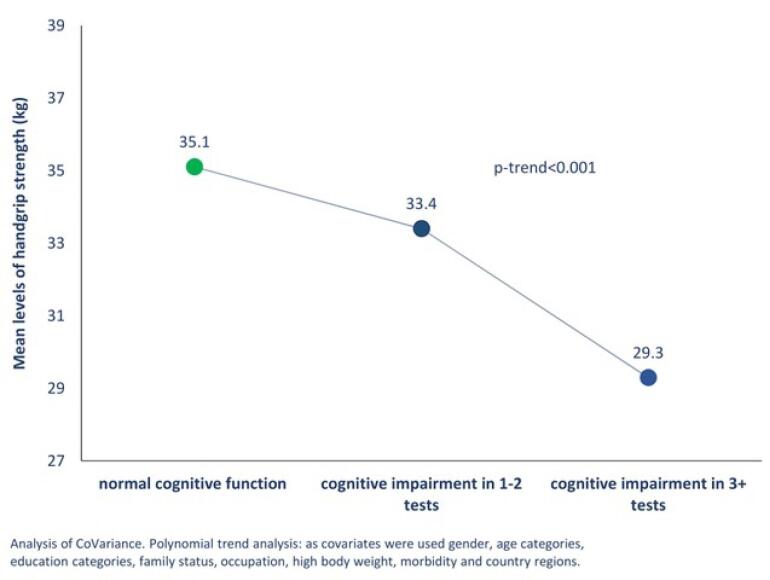
Handgrip strength levels of 41 395 Europeans aged 50 + participating in wave 8 of the SHARE survey (2019-2020) according to their cognitive function levels. *Note.* SHARE: Survey of Health, Ageing, and Retirement in Europe

Figure 2 illustrates that participants with cognitive impairment (3 + tests) had significantly lower mean levels of handgrip strength compared to those with cognitive impairment in 1-2 tests and those with normal cognitive function (29.3 *vs.* 33.4 and 35.1kg, respectively, *P* < 0.001). Physically inactive participants also had higher odds of cognitive impairment compared to those engaging in moderate/vigorous physical activity, both in 1-2 tests (Odds ratio [OR]:1.73, 95% CI: 1.32-2.26) and in 3 + tests (OR: 3.36, 95% CI: 2.57-4.40) (results not shown in the figure).

## Discussion

 The current study aimed to assess the cognitive function of European adults aged 50 + in relation to their handgrip strength and physical inactivity. It was found that approximately half of the participants have normal cognitive function, and more than 10% have cognitive impairment, the majority of which were females, without education or from Southern countries (*P* < 0.001). Moreover, higher levels of handgrip strength were observed in participants from Northern compared to Southern countries (*P* < 0.001), and more than 10% were physically inactive, while physical inactivity was more frequent in those with more conditions or in Southern countries (*P* < 0.001). In addition, participants with cognitive impairment (3 + tests) had lower levels of handgrip strength compared to those with cognitive impairment in 1-2 tests or with normal cognitive function (*P* < 0.001), and physically inactive participants had higher odds of cognitive impairment (*P* < 0.05).

 The recent systematic review by Pais et al^7^ reported that the prevalence of cognitive impairment worldwide ranges between 5.1% and 41.0%. In the same context and despite the methodological differences or criteria definitions, the present study determined that 13.3% of participants had cognitive impairments (3 + tests), representing an estimated population of 21 944 722 individuals. Although this figure may seem small, it is worrying due to the number of people who are potentially at real risk. Similarly, the Aging, Demographics, and Memory Study (ADAMS) showed a prevalence of cognitive impairment of 22.0% in people aged over 71 years, highlighting the importance of dementia as the predominant chronic degenerative disease.^23^ However, the qualitative evidence regarding the frequency of cognitive impairment indicated differences in terms of gender, education, and country. Cognitive impairment is more frequent in females than in males. A possible interpretation is the decrease in estrogen level at menopause or possibly a cognitive background in men that acts protectively against cognitive decline,^24^ or even higher life expectancy in females. Moreover, cognitive impairment is comorbid with diseases that more frequently affect women such as depression and increased risk of dementia, especially after menopause. This may also be due to the use of medication.^25,26^ Finally, females’ reduced access to education, compared to men, is related to reduced memory function.^27^ Education in this case seems to be a protective factor against cognitive decline.^28^

 Although the differences in handgrip dynamometry between genders seem to be established throughout one’s lifetime,^29^ its relationship with cognitive function must be assessed in the context of separating the genders or even within age groups. Another differentiation in cognitive impairment was related to European regions. Southern countries had almost three times higher prevalence of cognitive impairment (*P* < 0.001) compared to Northern countries, with physical inactivity and handgrip strength having a similar effect. Factors that may work protectively in this respect are the socioeconomic conditions of more developed countries, higher levels of educational and professional opportunities, lifestyle, and the promotion and encouragement of physical activity through good quality of health and life.^17^ Furthermore, the regional differences in the measurements of handgrip strength found between Northern and Southern European countries seem to be explained by genetic and environmental influences.^20^

 In the context of physical inactivity and sedentary lifestyles, which are significant behavioral risk factors for chronic diseases,^19,30^ the literature indicates that more than one-third of adults in European countries are classified as physically inactive, with this frequency reaching 45% in people aged 60 + .^19,31,32^ Studies have also displayed that Northern Europeans tend to be more physically active than their Southern counterparts, despite the subjective view of these nations being the opposite. This difference may be attributed in part to cultural diversity, lifestyle, climate differences in these countries, and possibly the sedentary behavior that seems to characterize Southern Europeans.^19,31,32^ Moreover, the organized environment in the Northern countries may encourage and motivate people to engage in higher levels of physical activity. Furthermore, the implementation of public health policy and the promotion of physical activity, by fostering such behavior, are the main focus of these countries. Overall, through weighted estimates, the current study showed that participants with cognitive impairment (3 + tests) have lower levels of handgrip strength compared to those with cognitive impairment in 1-2 tests or those with normal cognitive function. However, handgrip strength and physical inactivity were not controlled for in each of 5 test individually. Nevertheless, handgrip strength is associated with low processing speed and poor verbal or working memory.^13,33^ The measurement of handgrip strength in a clinical environment as part of routine patient assessment on admission can be used in various ways and will definitely benefit people with low handgrip strength, allowing them to receive other special interventions such as nutrition and physical activity.^34^

 Good cognitive function is intertwined with successful aging in the sense that adults lead an independent and high-quality life. It is, therefore, extremely important to identify people with MCI as it represents an excellent opportunity for intervention, aiming to reverse declining cognitive function via the appropriate treatment. It is, therefore, necessary to diagnose cognitive impairment accurately in the first place; since cognitive dysfunction takes several years or even decades to manifest, there is a maximum margin of the interception of the problem.^35^ Both in the clinical and the research field, it is highly important to include cognitive impairment and identify characteristics that can predict the imminent development of dementia.^28^ Appropriate interventions lead to potentially reversible forms of cognitive decline, starting with beneficial small changes in physical inactivity habits and lifestyle in general.^30^ Maintaining good physical condition and activity is important for healthy aging and functional independence, promoting and improving the quality of life for the elderly.

 A basic limitation of the current study is the causal documentation and interpretation of the relationship between cognitive function and handgrip strength and physical inactivity. These factors, however, are acquired over the lifetime (lifespan) of individuals or even through strong changes that have occurred in their lives (cohort effects).^36^ Nevertheless, the SHARE study, as a large-scale longitudinal study, not only offers recent data on European populations but also may be open to future evaluation. The findings of this study can help identify people already at increased risk of cognitive impairment, dementia, or Alzheimer’s disease. Furthermore, handgrip strength and assessment of physical inactivity can be useful diagnostic tools in clinical practice, especially when taking into account other risk factors such as hearing loss, insufficient nutrition, or medication use.^37^

**Highlights**

- The study confirms the relationship between physical exercise and cognitive function.
- Good physical condition and activity seem to be important for healthy aging, maintaining functional independence and improving the quality of life.
- The assessment of handgrip strength can be a useful diagnostic tool in clinical practice.

## Conclusion

 The SHARE research data of the present study in a European population aged 50 + found a low prevalence of cognitive impairment associated with low levels of handgrip strength and the presence of physical inactivity. This, therefore, indicates that these specific factors seem to play a significant role in early detection, diagnosis, and treatment, and slow down the progression and symptoms of cognitive impairment.

## Acknowledgments

 The authors would like to thank Dr. Rosemary Tzanaki for her valuable assistance in editing the manuscript.

## Authors’ Contribution

**Conceptualization:** Anastas Philalithis.

**Data curation:** Manolis Linardakis, Emmanouil Smpokos.

**Formal analysis:** Manolis Linardakis.

**Investigation:** Eleni Spiridaki.

**Methodology:** Manolis Linardakis, Nikos Rikos, Emmanouil K Symvoulakis, Ioanna Tsiligianni.

**Project administration:** Anastas Philalithis.

**Resources:** Manolis Linardakis.

**Software:** Manolis Linardakis.

**Supervision:** Manolis Linardakis, Nikos Rikos, Emmanouil K Symvoulakis, Ioanna Tsiligianni.

**Validation:** Manolis Linardakis, Nikos Rikos.

**Visualization:** Manolis Linardakis, Nikos Rikos.

**Writing–original draft:** Manolis Linardakis, Nikos Rikos, Emmanouil K Symvoulakis, Ioanna. Tsiligianni, Emmanouil Smpokos.

**Writing–review & editing:** Manolis Linardakis, Nikos Rikos, Emmanouil K Symvoulakis, Ioanna. Tsiligianni, Anastas Philalithis.

## Competing Interests

 The authors declare no conflict of interests.

## Ethical Approval

 The SHARE study was conducted according to the guidelines of the Declaration of Helsinki and approved by the Ethics Council of the Max Planck Society. All participants gave their informed consent to be included in the study.

## Funding

 SHARE survey, in all Waves, has several funding sources as referred to in the official site https://share-eric.eu/infrastructure/funding. 
